# Engineering an Autonucleolytic Mammalian Suspension Host Cell Line to Reduce DNA Impurity Levels in Serum-Free Lentiviral Process Streams

**DOI:** 10.1021/acssynbio.3c00682

**Published:** 2024-01-24

**Authors:** Geoffrey Howe, Matthew Wasmuth, Pamela Emanuelle, Giulia Massaro, Ahad A. Rahim, Sadfer Ali, Milena Rivera, John Ward, Eli Keshavarz-Moore, Chris Mason, Darren N. Nesbeth

**Affiliations:** † Department of Biochemical Engineering, University College London, Bernard Katz Building, London WC1E 6BT, United Kingdom; ‡ UCL School of Pharmacy, University College London, London WC1N 1AX, U.K.

**Keywords:** lentivirus, mammalian cells, bioprocess, gene therapy, nuclease

## Abstract

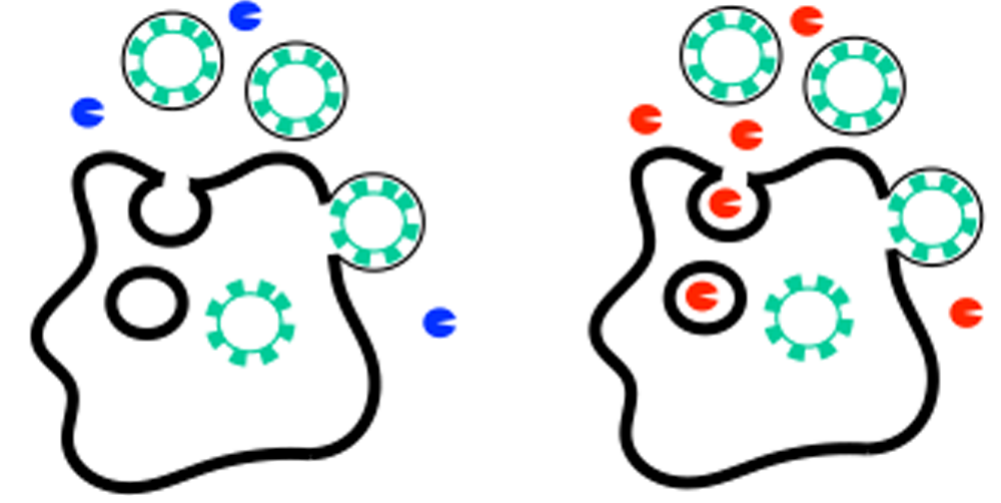

We engineered HEK293T cells with a transgene encoding tetracycline-inducible expression of a *Staphylococcus aureus* nuclease incorporating a translocation signal. We adapted the unmodified and nuclease-engineered cell lines to grow in suspension in serum-free media, generating the HEK293TS and NuPro-2S cell lines, respectively. Transient transfection yielded 1.19 × 10^6^ lentiviral transducing units per milliliter (TU/mL) from NuPro-2S cells and 1.45 × 10^6^ TU/mL from HEK293TS cells. DNA ladder disappearance revealed medium-resident nuclease activity arising from NuPro-2S cells in a tetracycline-inducible manner. DNA impurity levels in lentiviral material arising from NuPro-2S and HEK293TS cells were undetectable by SYBR Safe agarose gel staining. Direct measurement by PicoGreen reagent revealed DNA to be present at 636 ng/mL in lentiviral material from HEK293TS cells, an impurity level reduced by 89% to 70 ng/mL in lentiviral material from NuPro-2S cells. This reduction was comparable to the 23 ng/mL achieved by treating HEK293TS-derived lentiviral material with 50 units/mL Benzonase.

Lentiviral vectors have become an established gene therapy tool due to their ability to stably integrate active transgenes into the genomes of both dividing and nondividing cells.^1−5^ Lentivirus is often produced at scale via transient transfection of adherent mammalian cells using four plasmids, across which are safely distributed genes encoding virus structure, replication, infectivity, and therapeutic payload.^6−8^

Four-plasmid transfections introduce significant levels of DNA impurity into lentiviral process streams, necessitating DNA removal via adding high-cost commercial nucleases to the process stream^9−15^ and data capture for regulators demonstrating that DNA impurity levels are below a certain threshold.^16,17^ Furthermore, adherent mammalian cell growth is fundamentally constrained by the surface area of the horizontal containers in which they are grown. Fixed bed bioreactors, multilayer flasks, and microcarriers^18^ can enhance cell yield per volume but add significant production costs. The need to use bovine serum in growth media also brings a significant batch variation risk. These factors can contribute to the high prices of approved cell therapies, such as the $465,000 price point for Carvykti.^19,20^ Mammalian synthetic biology therefore has an opportunity to provide solutions to these bottlenecks in cell and gene therapy bioprocessing.

We previously demonstrated the feasibility of engineering adherent human embryonic kidney 293T (HEK293T) cells with transgenes encoding a secreted nuclease activity in a manner compatible with their use as hosts for lentivirus production.^21^ The logic for this approach in terms of the topology of the host cell interior and lentiviral budding is set out in Figure 1. Here we sought to re-engineer HEK293T with the ThorNucB transgene encoding *Staphylococcus aureus* NucB (UniProt: P00644) with the Semliki Forest virus capsid translocation signal for rapid egress^22^ and further improve the resulting cell line by adapting it to grow in suspension mode and in serum-free media. Finally, we sought to demonstrate the utility of this novel nuclease-engineered suspension cell line for reducing DNA impurities from lentiviral process streams.

**Figure 1:**
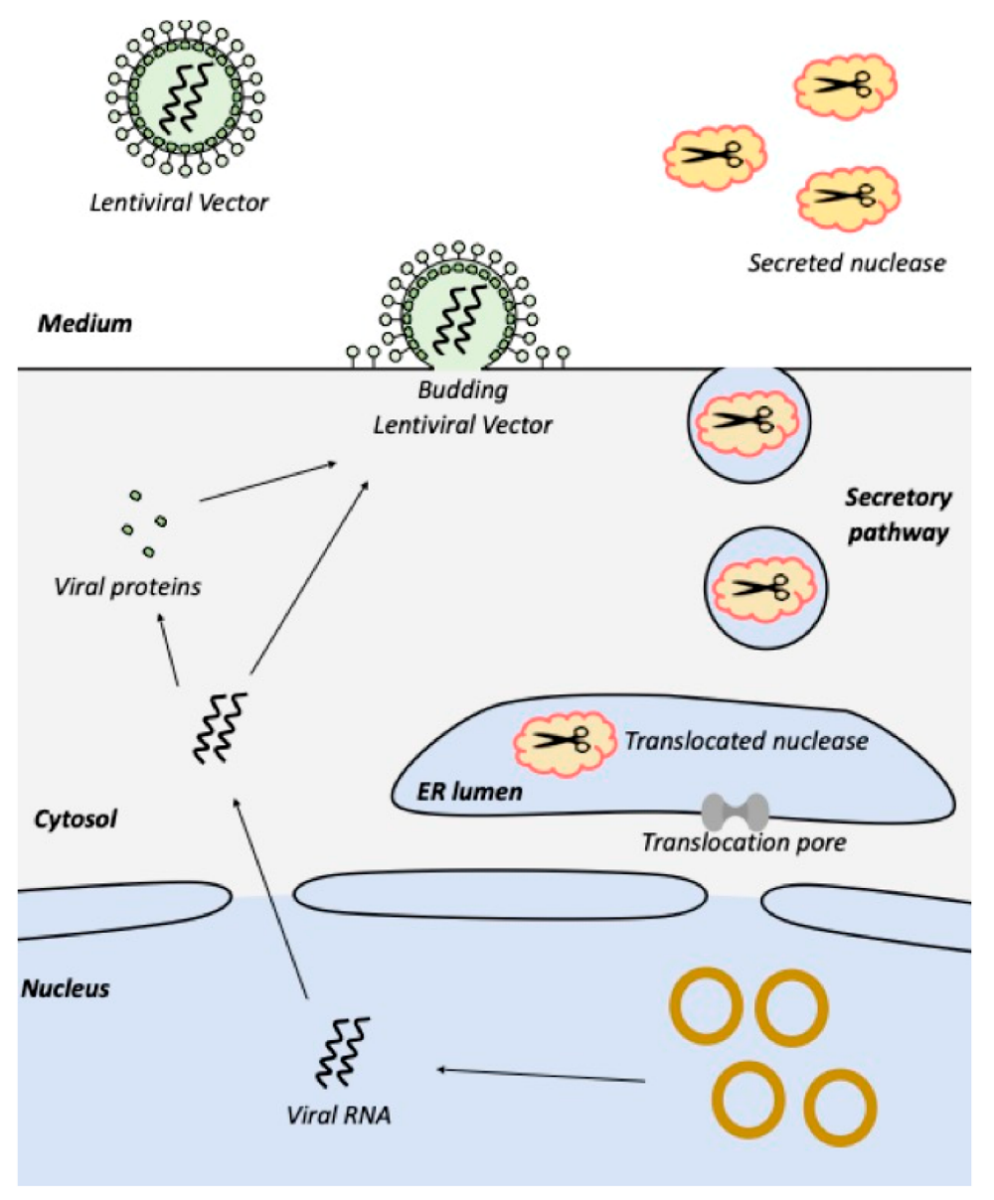
**Anticipated compatibility of nuclease secretion and lentivirus production.** Transgenes encoding lentiviral genomes and proteins are distributed across four plasmids (yellow circles) used for transient transfection of host cells. Complete lentiviral particles then assemble in the cytosol and, at the cell membrane, bud from the cell, taking a section of the membrane. Nuclease expressed from a genomically integrated transgene possesses a translocation signal intended to ensure that the recombinant enzyme is sequestered within the lumen of the endoplasmic reticulum (ER) and then trafficked in vesicles through the secretory pathway into the external milieu. Thus, host cell nucleic acids and lentiviral genomes are never in the same subcellular compartment as nuclease activity. Created with BioRender.

We stably transfected HEK293T cells with the pETIP-ThorNucB to generate the puromycin-resistant transformant cell line NuPro-2A. We then trialed three procedures for adapting adherent cells, conditioned for growth in 10% v/v FCS DMEM, to growth in suspension in the serum-free medium Freestyle293 (Figure 2A–G). We designed a five-step method in which cells would be passaged, once growth was observed, from 10% v/v FCS DMEM to 10% v/v FCS Freestyle293 and then subsequently to 5%, 2%, 1%, an 0.5% v/v FCS Freestyle293 in static T-flasks before a final transfer to 0% v/v FCS Freestyle293 in an agitated shake flask (Figure 2A). Using HEK293T cells, transfer to 5% v/v FCS Freestyle293 caused a drop to 75% viability and a drop in total cells, but both of these metrics returned to their previous levels in 10% v/v FCS Freestyle293. An initial drop in cell growth was repeatedly observed for the subsequent serum reduction steps, with cell growth finally increasing in serum-free Freestyle293 media in agitated shake flasks (Figure 2B), at which point 100% of cells were in suspension. For each transition to a lower serum percentage, all cells, both adherent and suspension, were pooled for each passage. Thus, we believe that a combination of the serum-free Freestyle293 medium, agitation, and use of a shake flask together favored the transition from adherent to suspension-mode growth, with the HEKT293T.5 cell line arising from this procedure.

**Figure 2:**
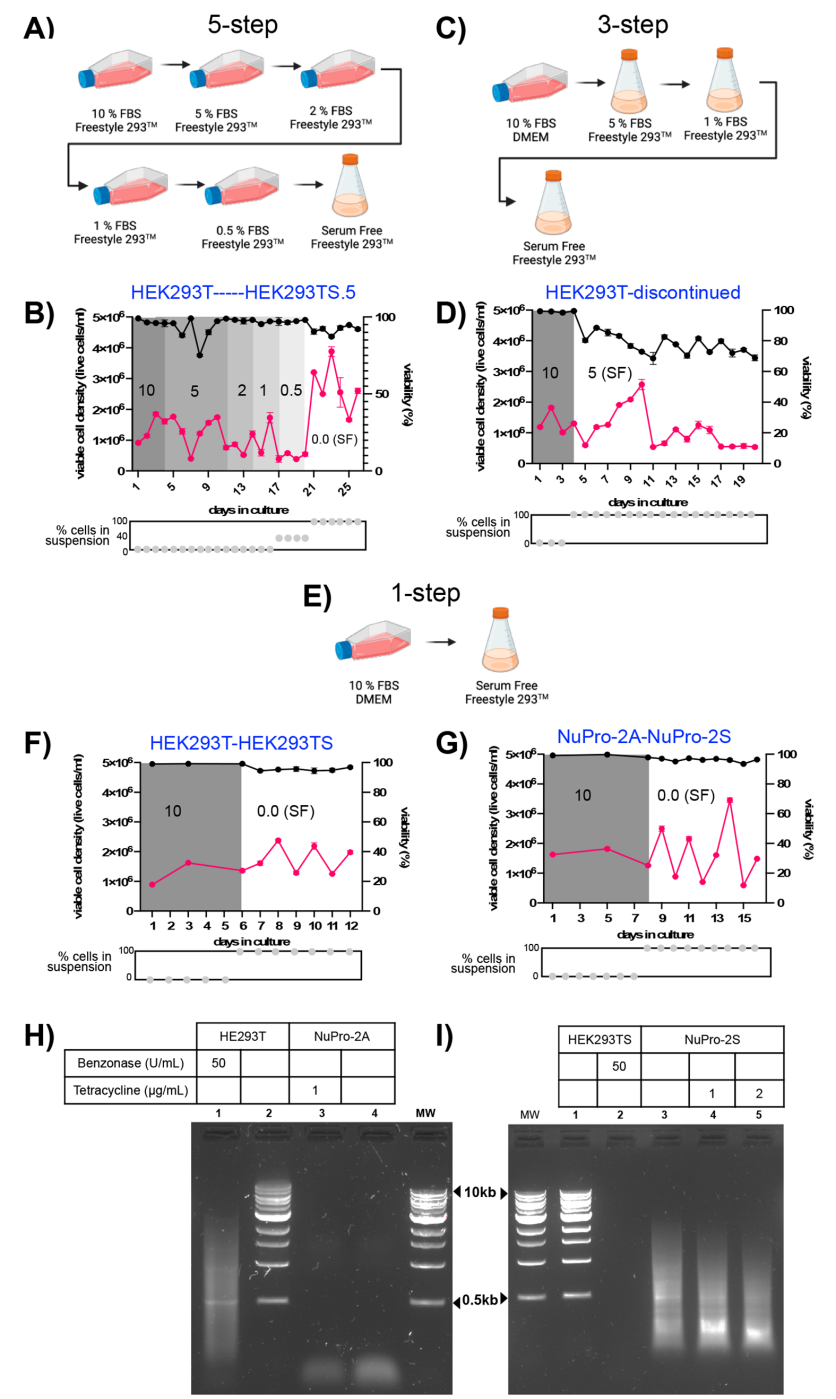
**Adapting adherent cells to serum-free growth in suspension.** (A) A five-step method in which v/v serum percentage was reduced stepwise in static culture before transfer to serum-free media in shake flasks. (B) Plots showing increase in viability metrics and suspension-mode growth such that the adapted cell line, HEK293TS.5, was established for ongoing cultivation. (C) Three-step method in which serum percentage would be halved to 5% v/v and cells transferred to shake flasks. (D) Plots showing suspension-mode growth and decrease in viability metrics such that the procedure was discontinued. (E) One-step method in which v/v serum percentage was reduced to zero and cells were transferred from static culture to a shake flask in a single step. (F) Plots of suspension-mode growth and increase in viability metrics such that the cell line HEK293TS was established. (G) Increase in viability metrics resulting in the adaptation of NuPro-2A into the cell line NuPro-2S. (H) Growth media samples from the indicated adherent cell lines, cultivated in 10% v/v FCS DMEM, were incubated with DNA ladder as described in Materials and Methods (lanes 2 and 4) or were additionally supplemented with Benzonase to 50 units/mL (lane 1), or the cell culture medium had been supplemented with tetracycline to 1 μg/mL 24 h prior (lane 3). (I) Growth media samples from the indicated suspension cell lines, cultivated in serum-free media, were incubated with DNA ladder alone (lanes 1 and 3) or with 50 units/mL Benzonase supplementation (lane 2) or for which the cell culture medium had been supplemented with tetracycline to 1 μg/mL (lane 4) or 2 μg/mL (lane 5) 24 h prior.

We next designed a three-step adaptation procedure that we hoped would work in a shorter time scale (Figure 2C). Notably, the transition from 10% v/v FCS Freestyle293 in a static T-flask to a lower serum level in Freestyle293 in an agitated shake flask again resulted in cells switching from adherent to suspension-mode growth. This first transfer also caused a persistent reduction in cell viability, and after 9 days in culture, cell growth also ceased (Figure 2D). In parallel we trialed a one-step procedure in which cells were transferred from 10% v/v FCS Freestyle293 in a static T-flask to zero-serum Freestyle293 in an agitated shake flask directly (Figure 2E). For both HEK293T (Figure 2F) and NuPro-2A (Figure 2G) cells, the transfer led immediately to suspension-mode growth and did not particularly impact viability percentage, and the total cell numbers underwent a period of variability before increasing at the end of the procedure.

The cell lines HEK293TS and NuPro-2S were derived from the parental HEK293T and NuPro-2A cell lines, respectively, using the one-step method. To confirm that the intended phenotypes had persisted through the adaptation process, we tested 10% v/v FCS DMEM from HEK293T and NuPro-2A cells for nuclease activity using a DNA ladder disappearance test (Figure 2H). For HEK293T cells, ladder disappearance occurred when Benzonase had been added to the media, but without this addition the ladder was unchanged (Figure 2H, lanes 1 and 2) compared to untreated ladder (Figure 2H, lane “MW”). Ladder disappearance occurred for media from NuPro-2A cells, whether tetracycline was added as an inducer or not (Figure 2H, lanes 3 and 4). For HEK293TS cells, in serum-free Freestyle293 media, nuclease activity was also absent (Figure 2I, lanes 1 and 2) unless provided by Benzonase. Serum-free Freestyle293 media from NuPro-2S cells showed clear ladder degradation in the absence of tetracycline, with this marginally increasing in response to the presence of two different tetracycline concentrations during cultivation (Figure 2I, lanes 3, 4, and 5).

After a cycle of cryopreservation and cryorevival to establish master cell banks, the growth performance of the HEK293TS (Figure 3A) and NuPro-2S (Figure 3B) was robust. Each cell line was used for transient transfection for lentivirus production (Figure 3C), with a Benzonase addition 2 h prior to harvest for HEK293TS and a tetracycline addition at the midpoint of virus production for NuPro-2S. The resultant titer performances of lentiviral material arising from the two procedures (Figure 3D,E) were not significantly different in terms of viral genomes/mL (vg/mL) and transducing units/mL (TU/mL).

**Figure 3:**
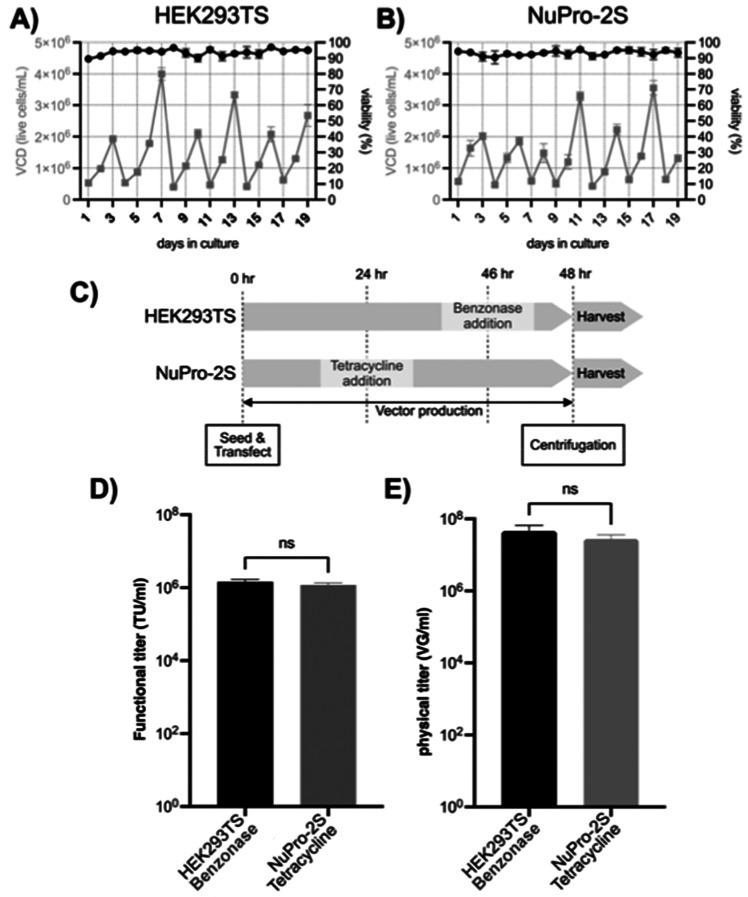
**Growth and lentiviral yield performance was unaffected by nuclease-engineering of host cells.** (A) HEK293TS and (B) NuPro-2S cell lines isolated in Figure 2 were cryopreserved and cryorevived using standard procedures, and their subsequent viable cell density (VCD) and viability (black and gray data points, respectively) were logged over six passages over 19 days postrevival. For both metrics, error bars are mean ± standard deviation (SD) of duplicate 20 mL shake flask cultures. (C) Both cell lines were used for lentivirus production in procedures that were matched except for addition of Benzonase to 50 units/mL concentration to HEK293TS cells 2 h prior to harvest and addition of tetracycline to 1 μg/mL concentration to NuPro-2S cells 24 h prior to harvest. (D) Lentivirus-containing growth media from both cell lines were used to transduce HEK293T target cells. and the resulting titers were plotted as transducing units/mL (TU/mL). Error bars are the combined mean ± SD of *n* = 3 biological repeats, which are 20 mL runs of lentivirus production by transient transfection, and *n* = 3 technical repeats, which are flow cytometric measurements on transduced cells of three individual wells. (E) Material tested in (D) was also used for RT-qPCR, and the resulting viral genomes/mL (vg/mL) were plotted. Error bars are the combined mean ± SD of *n* = 3 20 mL runs of lentivirus production and *n* = 2 RT-qPCR determinations. Differences in both TU/mL and vg/mL were not significant (ns) by unpaired Student’s *t* test.

During the transient transfection procedure characterized in Figure 3, 10 mL of HEK293TS culture was transferred to a 125 mL shake flask 2 h prior to harvest and had no Benzonase addition. At the 48 h harvest point (Figure 3C), for HEK293TS cells with and without Benzonase addition and for NuPro-2S cells, samples of the culture were removed for analysis and labeled “Whole Culture”. Further culture samples were removed and clarified by centrifugation, and the supernatant was retained and labeled “Clarified Media” (Figure 4A), which was the material used for titration in Figure 3D,E.

**Figure 4:**
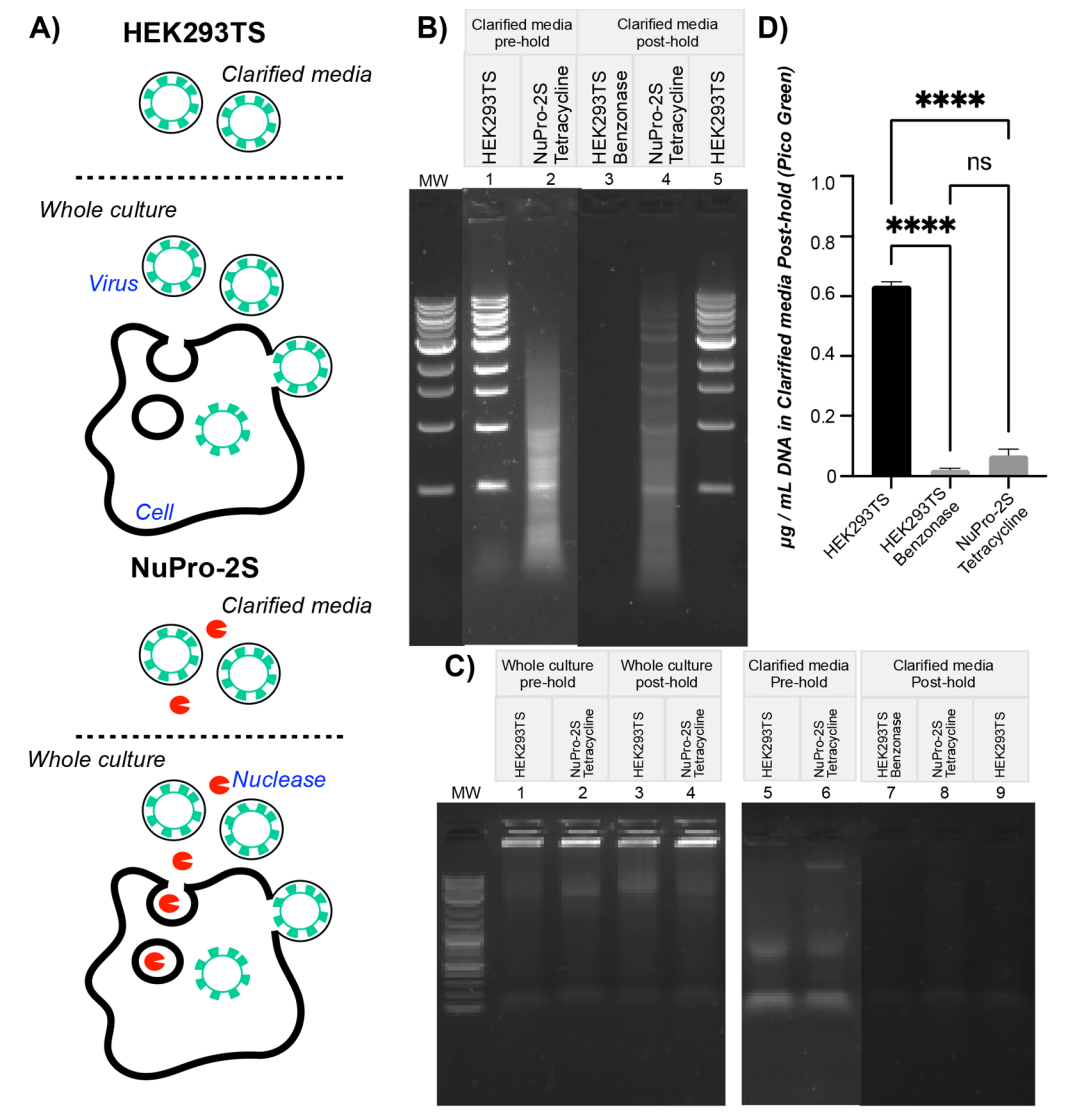
**During lentivirus production, NuPro-2S cells give rise to medium-resident nuclease activity that reduces DNA impurity levels *in situ*.** (A) Before and after the Figure 3C Benzonase addition, 10 mL samples of HEK293TS cells were centrifuged at 500 rcf for 5 min, and the supernatant was removed and retained as “Clarified Media”. Uncentrifuged samples were also taken and retained as “Whole Culture”. Equivalent NuPro2S samples were also taken. All samples were then incubated at 37 °C for a 1 h hold step. This diagram illustrates the anticipated presence of nuclease activity (red symbols) arising from NuPro-2S and the presence of lentivirus (green symbols) produced from both cell lines and confirmed in Figure 3. (B) “Clarified Media” samples (8 μL) were incubated with 2 μL of a 500 ng/μL 1 kb DNA ladder solution for 1 h prior to agarose gel electrophoresis. Samples were either pre- or posthold, as indicated, and taken from cultures of the indicated cell lines. Cultures had either had no additions or addition of tetracycline or Benzonase as detailed in Figure 3. (C) 40 μL samples, either pre- or posthold, from the indicated cultures were analyzed directly by agarose gel electrophoresis. (D) 100 μL serially diluted samples posthold from the indicated cultures were analyzed using the PicoGreen reagent, and the DNA was content plotted. Error bars represent the standard deviation of means arising from samples from *n* = 2 lentiviral production runs, each of which was used for *n* = 3 Pico Green-based determinations. Significance was determined by the unpaired Student’s *t* test. DNA concentration differences between unsupplemented HEK293TS and Benzonase-supplemented HEK293TS (*p* = 0.0921), and NuPro-2S (*p* < 0.0001) were significant (asterisks), while differences between Benzonase-supplemented HEK293TS and NuPro-2S were not (ns). All gel images are representative of duplicate gels used for analysis of duplicate samples.

Figure 4A illustrates our hypotheses with respect to clarified media: for HEK293TS cells, in addition to the lentivirus evidenced in Figure 3, it would contain no nuclease activity, as it had received no Benzonase addition; for NuPro-2S cells, in addition to lentivirus, there would be a nuclease activity arising from the engineered cells. In Figure 4B we tested this hypothesis by incubating “Clarified Media” from HEK293TS and NuPro-2S cells with the same mass of DNA ladder for 1 h. Significant degradation of ladder was observed for NuPro-2S (Figure 4B, compare “MW” lane with lane 2), whereas no degradation was observed for HEK293TS (Figure 4B, compare “MW” lane with lane 1). HEK293TS and NuPro-2S cells “Clarified Media” were then incubated at 37 °C for 1 h, while an additional HEK293TS sample was supplemented with Benzonase to 50 units/mL and incubated in the same manner for what we referred to as a “hold” step. For NuPro-2S (Figure 4B, lane 4) and HEK293TS (Figure 4B, lane 5) samples, the same pattern of nuclease activity was observed as prehold, with slightly less degradation in the case of NuPro-2S. Complete ladder digestion was observed in the Benzonase-supplemented sample (Figure 4B, lane 3).

Having confirmed the presence of nuclease activity in lentiviral material derived from NuPro-2S in serum-free Freestyle293 media, which received no nuclease additions, we next sought to determine the extent to which this nuclease activity was functional for reducing DNA impurity levels during lentiviral processing. Agarose gel electrophoresis with SYBR Safe staining revealed no obvious difference in DNA levels between “Whole Culture” samples pre- and posthold (Figure 4C, lanes 1–4). For NuPro-2S and HEK293TS samples, some DNA is visible in the prehold “Clarified Media” samples (Figure 4C, lanes 5 and 6), with a diffuse band of approximately 10 kb being visible for NuPro-2S, while a band of similar intensity was present in the sample well for HEK293TS material.

After the 1 h hold step, HEK293TS, Benzonase-supplemented HEK293TS, and NuPro-2S cells “Clarified Media” samples all showed less DNA than the prehold sample, with any differences between the gel lanes being difficult to discern (Figure 4B, lanes 7–9). We next used a Quant-iT PicoGreen dsDNA Assay Kit to measure DNA content in these posthold samples (Figure 4D). For HEK293TS samples, PicoGreen determined that 635.09 ng/mL DNA was present. Benzonase supplementation reduced this DNA to 23.09 ng/mL, while material from NuPro-2S cells with no Benzonase addition contained only 69.89 ng/mL DNA, an 89% reduction in DNA impurity content compared to untreated lentiviral material from HEK293TS cells.

The field of mammalian synthetic biology continues to foster innovative approaches to cell engineering^23^ which are being increasingly applied to improve manufacturability^24^ and clinical efficacy^25^ of a variety of gene therapy tools.^26^ The data presented here prove that HEK293T cells, often the workhorse of commercial lentivirus production, can be rapidly adapted to grow in suspension mode and in serum-free media. HEK293T cells thus adapted can also be engineered with transgenes such that nuclease activity can be detected in their growth media that is sufficient to cause an 89% reduction in the level of DNA impurity arising from lentivirus production, a level of reduction which would otherwise require costly Benzonase supplementation to achieve.

## Materials and Methods

### Plasmid Handling

Standard molecular biology techniques were used for all plasmid propagation, isolation, and analytical procedures. The plasmid pETIP-ThorNucB^21^ encodes the *S. aureus* nuclease (NucB) with its native translocation signal replaced with an influenza hemagglutinin translocation signal peptide fused directly to an influenza hemagglutinin eptitope tag (HAss-HAtag) followed by a 62-residue region of the amino terminal domain of the Semliki Forest Virus capsid protein (Cp-p62). The following third-generation lentivirus plasmids were used: pLJM1-eGFP (Addgene plasmid no. 19319) encoding the eGFP reporter payload genome, pMDLg/pRRE (Addgene plasmid no. 12251) encoding gagpol proteins, pRSV-Rev (Addgene plasmid no. 12253) encoding rev, and pMD2.G (Addgene plasmid no. 12259) encoding the VSV-G envelope protein.

### Mammalian Cell Cultivation

HEK293T cells, obtained from the American Type Culture Collection (ATCC) (cat. no. CRL-3216), and NuPro-2A cells were maintained in Dulbecco’s modified Eagle’s medium (DMEM) (Gibco, UK) supplemented with 10% heat-inactivated fetal bovine serum (FBS) (Gibco, UK). HEK293TS.5, HEK293TS, and NuPro-2S cells were maintained in Freestyle293TM medium (Thermo Fisher Scientific, UK) in 125 mL vent cap cell culture flasks (Corning, UK). All adherent cell lines were cultivated in Nunc Cell-Culture Treated T-flasks (Thermo Fisher Scientific, UK) and passaged every 48–96 h at 37 °C with 5% CO_2_.

### Stable Transfection of HEK293T cells with pETIP-ThorNucB

Stable HEK293T transfection was performed using 10 μg of pETIP-ThorNucB plasmid^21^ and Fugene6 transfection reagent (Promega, UK) as per manufacturer’s instructions, followed by selection using 3 μg/mL puromycin dihydrochloride (Thermo Fisher Scientific, UK) 4 days post-transfection.

### Detection of Nuclease Activity by DNA Ladder Disappearance

Nuclease activity was identified by adding 8 μL of a specified sample to 2 μL of a 500 ng/μL solution of 1 kb DNA ladder, 500 bp–10 kb, from New England Biolabs (NEB), at 37 °C for 1 h (Figures 2 and 4), which was dissolved in nuclease-free water (Invitrogen). Postincubation, reactions were halted by adding an EDTA-containing 6× loading dye (NEB, UK) and subsequently analyzed by electrophoresis in a 1% w/v agarose gel. This gel contained SYBR Safe DNA Gel Stain (Thermo Fisher Scientific, UK) and utilized tris(borate) EDTA (TBE) as both gel solvent and running buffer. Growth medium samples were created by centrifuging the harvested growth media at 500 rcf for a duration of 5 min. The supernatant was gently moved to a fresh centrifuge tube before use in this procedure.

### Lentivirus Production

Lentivirus was produced by transient transfection of suspension, serum-free-adapted HEK293TS and NuPro-2S cell lines in a procedure informed by Bauler et al.^9^ Briefly, 20 mL of cells were seeded at 2 × 10^6^ live cells/mL in 125 mL of nonbaffled vent cap cell culture flasks (Corning, UK). A solution containing 22 μg of DNA was formulated, consisting of the four plasmids pLJM1-eGFP, pMDLg/pRRE, pMD2.G, and pRSV-Rev at a mass ratio of 56:16:8:1, respectively. This DNA solution was brought to 1000 μL by the addition of OptiMEM (Thermo Fisher Scientific, UK). A 1 mL solution of 22 μL PEIpro transfection reagent (Polyplus Transfection, France) was added to a DNA solution containing 22 μg total of plasmid DNA then made up to 1 mL solution total with OptiMEM and incubated for 15 min at room temperature. This PEI/DNA mixture was added to cells dropwise.

48 h post-transfection, the entire cell culture was transferred into sterile 50 mL tubes and centrifuged at 500 rcf for 5 min (5920 R centrifuge with S-4x1000 swinging bucket rotor; Eppendorf, UK). The supernatant was removed by careful decanting and frozen at −80 °C (CryoCube F570n, Eppendorf, UK) prior to analysis or purification.

### Lentivirus Transduction

HEK293T target cells were coseeded at 6 × 10^4^ cells per well in 96-well plates in 30 μL of 10% v/v FCS DMEM with 10 μg/mL Polybrene (Merck Life Science UK Limited, UK) alongside addition of 20 μL of serially diluted lentiviral material. Twenty-four hours postseeding, 100 μL of fresh, prewarmed 10% v/v FBS DMEM was added to each well, and 48 h later cells were harvested, fixed in 4% v/v paraformaldehyde (Alfa Aesar, UK), and immediately analyzed by flow cytometry (BD LSRFortessa, BD Biosciences, UK). Nontransduced cells were used to normalize the percentage of GFP-expressing cells for calculating transducing units per mL (TU/mL) as follows:
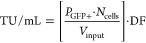
where *P*_GFP+_ is the percentage of GFP-expressing cells, *N*_cells_ is the number of seeded target cells at transduction, *V*_input_ is the vector input volume, and DF is the dilution factor applied to the volume of lentiviral material. All analysis of flow cytometric data was performed using FlowJo v10.7.1 (BD Biosciences, UK) software. Appropriate gates were applied to isolate HEK293T cells, singlets, and GFP-expressing cells. For analysis, a minimum of 10,000 events were recorded.

### Real-Time qPCR

Viral RNA was isolated from lentiviral material using the QIAamp Viral RNA Kit (QIAGEN, UK) as per manufacturer’s instructions. Twenty-five microliters of extracted RNA was added to 2 μL (4 units) of RNase-free DNaseI (NEB, UK), 5 μL of 10× DNase I reaction buffer (NEB, UK), and 18 μL of nuclease-free water (Corning, UK) in a final reaction volume of 50 μL, and the mixture was incubated for 30 min at 37 °C to remove any DNA from the sample. DNaseI was then inactivated by incubation at 75 °C for 10 min. Five microliters of serially diluted RNA sample was then used as template for RT-qPCR using the iTaq Universal SYBR Green One-Step Kit (Bio-Rad, USA) with an eGFP amplicon primer pair of TACTGACGCTCTCGCACC and TCTCGACGCAGGACTCG^28^ in a 20 μL total reaction volume. Vector genomes per milliliter (vg/mL) was calculated by comparing cycle threshold (Ct) values against Ct values of a standard curve generated using serially diluted StemMACS EGFP mRNA of known concentration (Miltenyi Biotec, Germany).

### Measuring DNA Content in Whole Cell and Growth Media Samples

Whole cell and growth media samples (Figure 4C) were analyzed by electrophoresis within a 1% w/v agarose gel stained with SYBR Safe DNA Gel Stain (Thermo Fisher Scientific, UK) using tris(borate) EDTA (TBE) as the gel solvent and running buffer. “Whole Culture” samples of typically 1 mL were taken directly from a given growth vessel. “Clarified Medium” samples were prepared by centrifugation of harvested “Whole Culture” samples at 400 rcf for 5 min. Supernatant was then carefully transferred to a new centrifuge tube for use in this procedure. Total double-stranded DNA (dsDNA) was measured using the Quant-iT PicoGreenTM dsDNA Assay Kit (Thermo Fisher Scientific, UK). Samples were serially diluted to within the range of the standard curve and assayed as per the manufacturer’s instructions. Relative fluorescence units (RFU) were compared against a standard curve composed of serially diluted lambda DNA to calculate the amount of DNA in nanograms per milliliter in the original samples.
